# Isobornyl and Isocamphyl Photostabilizers in Poly(lactic acid)-Based Electrospun Fibers

**DOI:** 10.3390/polym16060855

**Published:** 2024-03-20

**Authors:** Vladimir Belyi, Ivan M. Kuzivanov, Irina Fedorova, Olga A. Shumova, Nikita Paderin, Pavel A. Markov, Ilya I. Pikovskoi, Irina Yu. Chukicheva, Alexander V. Kutchin

**Affiliations:** 1 Institute of Chemistry, Federal Research Center “Komi Scientific Center”, Ural Branch, Russian Academy of Sciences, 48 Pervomayskaya Str.,167982 Syktyvkar, Russiachukicheva-iy@chemi.komisc.ru (I.Y.C.);; 2 Institute of Physiology of Federal Research Centre “Komi Science Centre of the Urals Branch of the Russian Academy of Science”, 167982 Syktyvkar, Russia; paderin_nm@mail.ru; 3 National Medical Research Center for Rehabilitation and Balneology, 121099 Moscow, Russia; p.a.markov@mail.ru; 4 Core Facility Center “Arktika”, M.V. Lomonosov Northern (Arctic) Federal University, Northern Dvina Emb. 17, 163002 Arkhangelsk, Russia; i.pikovskoj@narfu.ru

**Keywords:** renewable raw materials, polylactide, photoprotectors, glass transition, Py–GC/MS

## Abstract

In this work, electrospun polylactide fibers with new photostabilizing additives, 4-methyl-2,6-diisobornylphenol (DIBP) and N-isocamphylaniline (NICA), have been tested under the influence of UV-C radiation (254 nm). The changes in the polymers’ chemical structure under UV-C radiation were revealed through the increase in absorption in the 3600–3100 cm^−1^ region in regard to the FTIR spectra. In the samples that were irradiated for 1 h, the stabilizing effect of the photoprotectors became most noticeable as the difference in the content of the hydroxyl groups in stabilized and the pure PLA reached a maximum. The TG–DSC method revealed that the most sensitive indicator of the irradiation effect was the glass transition temperature (T_g_), which persisted after 2 h of irradiation when using photostabilizers and their combinations. The PLA/DIBP(1) and PLA/NICA(1) samples showed the best results in protecting PLA from UV-C radiation based on the T_g_ values; although, the mixture of DIBP and NICA was not as effective. The chemical structure of the photostabilized PLA samples was studied using NMR, GPC, and Py–GC/MS analysis. The electrospun polylactide fibers were mechanically tested and the effects of the electrospun samples on cell viability were studied.

## 1. Introduction

Polymers quite often undergo photo-oxidative degradation during use in sunlight or near germicidal ultraviolet sources. When polymer units are exposed to UV radiation, a hydrogen atom is abstracted to form a radical that causes a chain-oxidation reaction involving available oxygen. Polymer chains can also be cleaved via direct photolysis of intramolecular bonds, by high-energy photons. The reduction in molecular weight of polymers results in declining strength and ductility, and sometimes discoloration and texture changes occur. The sensitivity of polymers to the photo-oxidative impact varies, depending on the characteristics of the chemical structure. Unfortunately, most polymers used in construction materials and everyday life are very sensitive to UV radiation [1].

Photo-oxidative degradation can be slowed down by protective additives. Photostabilizers can absorb light, releasing the absorbed energy as heat, and may take part in chemical reactions with decomposition products, or slow down undesirable processes. Hindered phenols or organophosphorus compounds can be used as antioxidants that interrupt the action of free radicals. Hindered amines shield the polymer by absorbing UV radiation, and they also deactivate hydroperoxides and excited chromophores.

Polylactide (PLA) is a biocompatible aliphatic polyester derived from lactic acid, obtained through the enzymatic processing of plants [2,3,4]. This polymer is now widely in demand in 3D printing, medicine, and as a packaging and covering material [5,6]. However, it is known that pure PLA is highly susceptible to UV radiation, so the issue of photodegradation of new composites and PLA blends is being actively studied [7,8,9,10,11]. UV radiation does not penetrate deep into PLA, but can affect the surface layers of the polymer, for e.g., reducing the molecular weight, increasing polydispersity, and initiating cross-linking and chain-termination chemical transformations [12,13], which seems especially important for materials obtained by electrospinning, due to their fine structure and high surface area. The modulation of the UV-resistant properties of PLA materials is in demand in medicine, where polymers can be affected by ultraviolet germicidal irradiation [14].

Terpenophenols are a relatively new class of compounds based on phytochemicals. Physicochemical and biological studies have shown that terpenophenols exhibit higher antioxidant activity compared to butylated hydroxytoluene (BHT), and it was noted that the toxicity of terpenophenols was significantly lower than that of BHT [15,16,17]. However, it is unknown whether terpenophenols preserve the elasticity and strength of electrospun polymer materials with a large surface area. Our previous study [18] showed that 2-isobornylphenol exhibits good photoprotective properties, but has poorer thermo-oxidative protection compared to a similar aromatic amino terpene, N-isobornylaniline. PLA films were found to be protected from cracking after 4 h of irradiation [18].

**Figure 1:**
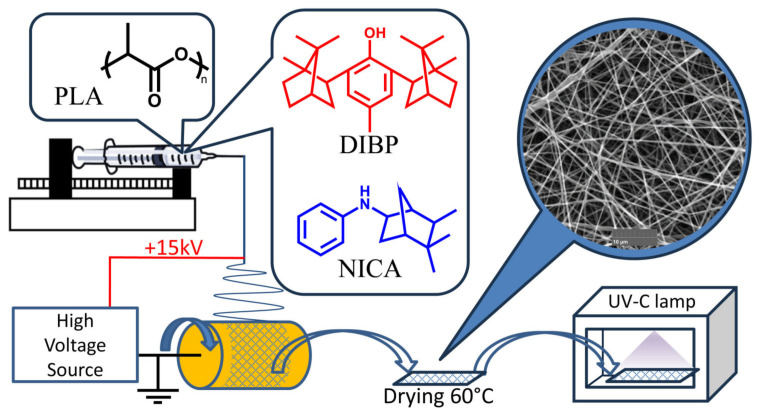
Preparation and testing of PLA materials with the photostabilizers: 4-methyl-2,6-diisobornylphenol (DIBP) and N-isocamphylaniline (NICA).

The introduction of an additional terpene moiety into the 2-isobornylphenol structure is considered as an additional stabilizing factor for the phenoxyl radical in the polymer photoprotective activity. This was tested in the present study, using the example of 4-methyl-2,6-diisobornylphenol (DIBP) in comparison with N-isocamphylaniline (NICA) (Figure 1). These new terpene-based additives were used as photostabilizers for the first time in the composition of electrospun PLA materials against exposure to UV-C radiation, with a wavelength of 254 nm. The effect of the structural features of the new stabilizers on their activity was studied using FTIR, UV, NMR, GPC, TG–DSC, and Py–GC/MS methods. The electrospun polylactide fibers were mechanically tested and the effects of the electrospun samples on cell viability were studied.

## 2. Materials and Methods

### 2.1. Materials

In this study, the PLA Ingeo™ Biopolymer 4043D, manufactured by NaturalWorks (Blair, NE, USA), was used. Dichloromethane (CH_2_Cl_2_, 99,8%, PJSC ”Khimprom”, Novocheboksarsk, Russia) and dimethyl sulfoxide (DMSO, 99%, "Component-Reagent", Moscow, Russia) were used. The substances studied as photostabilizers (see Figure 1), 4-methyl-2,6-diisobornylphenol (DIBP) and N-isocamphylaniline (NICA), were synthesized using the methods described in [19,20]. The UV spectra of DIBP, NICA, and PLA were obtained at UV-1700 (Shimadzu, Kyoto, Japan) in chloroform solution, in the range of 200–600 nm.

### 2.2. Formation of Electrospun Fiber Mats

The electrospun poly(lactic acid)-based fiber mats with added DIBP and NICA were obtained by electrospinning PLA solutions, pumped with a syringe dispenser from the blunt tip of a stainless steel needle (i.d. = 0.2 mm), to which an electric field was applied (Figure 1).

The polymer solutions were as follows: the concentration of PLA in a 4/1 *v*/*v* mixture of CH_2_Cl_2_/DMSO was 7% *w*/*v*. The calculated mass of DIBP and NICA were dissolved in these solutions to prepare the following compositions (Table 1).

The polymer solutions were electrospun on aluminum foil covering the rotating grounding electrode (d = 50 mm, l = 100 mm, 60 rpm). The parameters were as follows: the solution feed rate was 0.5 mL·h^−1^, the voltage on the needle was +15 kV, the distance between the needle and the grounding electrode was 100 mm, and the electrospinning period was 30 min.

The obtained electrospun PLA/DIBP and PLA/NICA samples were dried stepwise at 40 °C, 50 °C, and 60 °C, with 10 min of exposure in air at each temperature.

### 2.3. Irradiation of Electrospun Fiber Mats

The irradiation was carried out in a 300 × 160 × 400 mm chamber, with a radiation source inside, namely a quartz lamp, ESL-PLD-25/UVCB/E27/CL (Uniel Lighting Co. Ltd., Hangzhou, China), with a maximum radiation of 254 nm, a power of 25 W, and a temperature of 29 °C. The irradiation time intervals were 15 min, 30 min, 1 h, 2 h, 4 h, and 8 h. The distance between the irradiated sample and the radiation source was 40 mm, and the incident light intensity was 46 mW cm^−2^.

### 2.4. Characterization of Electrospun Fiber Mats

To study the morphology of the surface and cracks in the irradiated polymer samples, scanning electron microscopy was used, with a Vega3 SBU (TESCAN) microscope (Brno, Czech Republic). The samples were previously coated with gold, with the use of a sputter coater DSCR (Nano-Structured Coatings Co., Tehran, Iran), to increase their conductivity. The sputtering thickness was 40 angstroms. The following conditions were used for the SEM: elastic electron scattering mode, accelerating voltage of 10 kV.

### 2.5. TG–DSC Analyses

Simultaneous thermal analysis (thermogravimetry and differential scanning calorimetry) was performed using a STA 409 PC Luxx (“NETZSCH”, Selb, Germany) device, in dynamic temperature mode. A weighed sample (about 10 mg) was placed in an aluminum crucible, with a diameter of 5 mm, without a lid; a similar empty crucible served as a reference sample. Thermograms were obtained in an air atmosphere (20 mL/min), in the temperature range 25–400 °C, at a heating rate of 10 K/min. Thermogram analysis was carried out using the software package NETZSCH Proteus Thermal Analysis (version 4.8.5). Each sample was studied in 2–3 repeats.

### 2.6. FTIR Analyses

Fourier transform infrared spectroscopy (FTIR) of the original and irradiated PLA sample was performed with an IR Fourier spectrometer IR Prestige-21 (Shimadzu, Kyoto, Japan), equipped with a DLATGS detector, to analyze changes in the functional groups and bonds caused by UV-C irradiation. The transmission spectra were obtained in the diffuse reflection mode. Spectra were recorded in the range of 4000–700 cm^−1^ at a resolution of 4 cm^−1^, and the number of scans was set to 20. The data were processed using the software IRsolution Version 1.30 (Shimadzu, Kyoto, Japan). The spectra were integrated quantitatively using Origin 6.1.

### 2.7. NMR Analyses

The ^1^H and ^13^C JMOD NMR spectra of the PLA samples, before and after irradiation, were recorded in 5 mm tubes by a Bruker Avance II 300 spectrometer (Billerica, MA, USA). Around 0.1 g of the polymer sample was dissolved in 0.6 cm^3^ of deuterated chloroform (CDCl_3_, >99.9%, Solvex). The spectra were referenced in regard to the residual signals of chloroform (7.26 ppm for ^1^H and 77.5 ppm for ^13^C spectra). The spectra were processed using the Spinsolve 1.19.2 program.

### 2.8. Gel Permeation Chromatography

The average molecular weight (M_w_) and molecular weight distribution of the samples and their changes during the degradation tests were analyzed using gel permeation chromatography (GPC) by the LC-20 Prominence HPLC system (Shimadzu, Japan), equipped with a RID-20A refractive index detector. The samples were dissolved in stabilized THF (~1 mg mL^−1^). A series of SDV stationary phases (10,000 Å, and 1,000,000 Å) (300 × 7.8 mm, PSS, Esslingen, Germany) were used for the separation. The analyses were carried out at 30 °C in THF during the mobile phase, with a 0.8 mL min^−1^ flow rate, and a loading volume of 40 mL. The GPC system was calibrated with standard monodisperse samples of polystyrene standards, ranging from 1000 to 1,300,000 g mol^−1^ (PSS, Germany). The molecular weight averages (M_w_, M_n_) and molecular weight dispersity index (MWD, M_w_/M_n_) were calculated with the aid of polystyrene standards, based on a “universal” calibration curve. All data processing were carried out using the GPC LabSolution (Version 5.71 SP1, Shimadzu, Japan) software.

### 2.9. Py–GC/MS Analyses

The irradiated PLA samples were pyrolyzed with the use of a multifunctional pyrolyzer, EGA/PY-3030D (Frontier Lab., Kooriyama, Japan), connected to a gas chromatograph, coupled with a mass selective detector (GCMS-QP2010Plus, Shimadzu, Japan). The irradiated PLA samples were pyrolyzed at 600 °C for 0.2 min. The interface temperature was 300 °C.

The DB-5ms column used for the analysis was 60 m long (0.25 um thickness, 0.32 mm diameter); using helium as the carrier gas, with a 100:1 split ratio. The gas chromatograph oven was programmed at 40 °C for 1 min, followed by an increase of 20 °C min^−1^ up to 300 °C, with a final isotherm for 30 min. The mass selective detector was programmed to detect the m/z diapason of 29–600. The identification of PLA pyrolysis products was conducted from the obtained Py–GC/MS spectra using NIST08 databases.

### 2.10. Texture Characterization

The electrospun PLA materials were studied using the puncture test. The test was carried out using a TA-XT Plus (Texture Technologies Corp., Stable Micro Systems, Godalming, UK) instrument at room temperature, equipped with a P/2 probe, with a diameter of 2 mm, a depth of 6 mm, and 1.0 cm high.

### 2.11. Cell Morphology

To assess the effects of the samples on cell viability, co-incubation of the examined samples with human fibroblasts was performed (Cell Applications, San Diego, CA, USA, Cat. No. 106 K-05a). To characterize the cell viability, the samples (ø5 mm) were sterilized with a 70% alcohol treatment and UV irradiation, and then placed into a 96-well plate, one sample per well, with a total of five replicates for each sample. An aliquot of 0.1 mL of the cell culture medium (DMEM, 10% FBS, 1% PS), containing fibroblasts (2 × 10^4^ cells/mL), was added to the wells.

Culturing was performed in standard conditions (37 °C, 5% CO_2_). Five days after the joint incubation of the examined samples with human fibroblasts, the morphometric characteristics of the fibroblasts were evaluated. A Leica DMI4000 microscope (Leica Microsystems, Wetzlar, Germany) and methods involving bright-field, polarization, and fluorescent observation were used to assess the morphometric characterization of the cells. To improve fibroblast visualization, the cells were fixed in 70% ethanol and stained with a rhodamine dye.

### 2.12. Statistical Analysis

The statistical analysis was performed using the statistical software BioStat (version 4.03) and Microsoft Office Excel 2007. All experiments were performed, at least, in triplicate. The results are presented as the mean ± standard deviation (SD). Multiple comparisons were performed with a one-way ANOVA and Tukey’s HSD test. A probability value of *p* < 0.05 was considered statistically significant.

## 3. Results and Discussion

### 3.1. Texture Characterization

**Figure 2:**
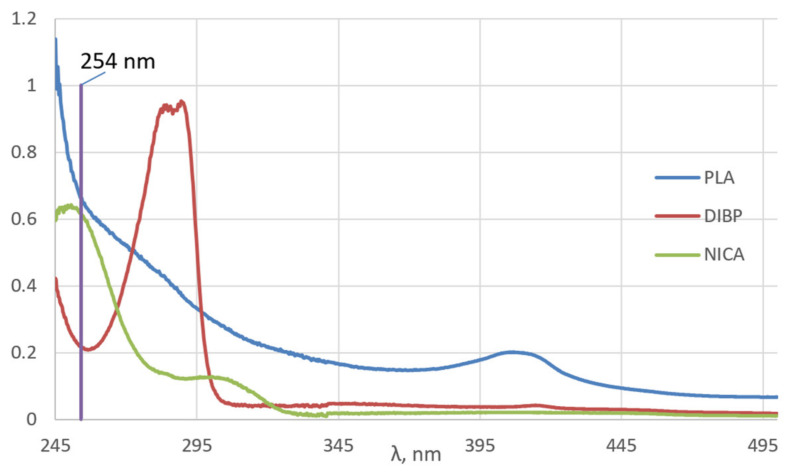
UV absorption spectra of the original PLA, DIBP, and NICA.

Short-wave radiation, in the wavelength range 100–280 nm (UV-C), is the most damaging type of UV radiation for polymer materials. Figure 2 shows the UV absorption spectra of the original PLA, and DIBP, NICA. It is known that UV radiation in the range of 220–280 nm is absorbed by the PLA carbonyl group and causes an n-π* electronic transition. This excited state of the electron is capable of inducing a macromolecular chain-breaking reaction, according to the Norrish I and Norrish II mechanisms [3,21]. These reactions result in a decrease in the molecular weight of macromolecules, an increase in polydispersity, and the appearance of carboxylic, acetic anhydride, and allylic groups.

As for additives to polymers, DIBP belongs to a group of UV stabilizers of the class of sterically hindered phenols, and NICA is a light stabilizer of the class of hindered amines. The UV spectrum of DIBP is characterized by an absorption maximum of 289 nm and a minimum of 256 nm. Judging by the UV spectrum, DIBP is unable to shield the polymer against the 254 nm UV radiation used in this work. For sterically hindered phenols, such as DIBP, it is assumed that in the first stage of stabilization, the hydrogen atom in the phenolic group is transferred to the radical species that arise in the polymer. At the second stage, a hydrogen atom in the α-position is donated, which leads to the formation of a quinone structure [22].

NICA has an absorption maximum of 253 nm, which practically coincides with the UV range used in this work. It is known that the photostabilizing ability of hindered amines is based on the formation of nitroxide radicals under the influence of radiation, which are able to enter into a recombination reaction with a polymer radical and, therefore, interrupt destructive processes in the polymer chain [23].

**Figure 3:**
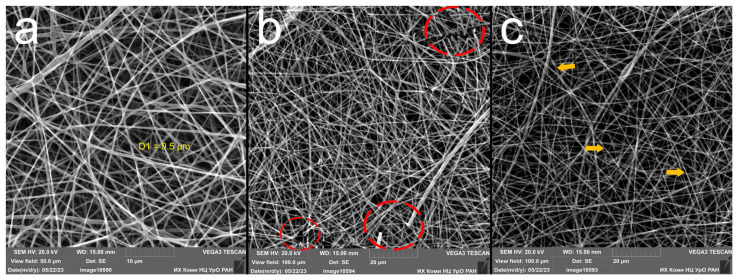
Microphotographs of the electrospun PLA fibers: nonirradiated PLA (**a**), 30 min of irradiation (**b**), 1 h of irradiation (**c**).

Figure 3a shows pristine electrospun PLA fibers, which has a diameter of about 0.5 μm. Changes in the strength of PLA fibrous materials without stabilizer additives become noticeable in samples irradiated in the UV-C range for 30 min, namely the film retains its integrity with light manipulation with tweezers, but becomes brittle under stretching or bending. Figure 3b shows the sites of the ruptures (red circles) in the electrospun material irradiated for 30 min. However, in the samples with the addition of DIBP and NICA, and their mixtures, PLA/(DIBP + NICA)(0.1) and PLA/(DIBP + NICA)(1), the elasticity and strength were maintained after 30 min of irradiation. The PLA/DIBP(1), PLA/DIBP(0.1), and PLA/NICA(1) samples irradiated for 1 h, as well as the PLA/(DIBP + NICA)(0.1) and PLA/(DIBP + NICA)(1) samples, became noticeably more fragile, but they could be removed from the foil as a single sheet. The PLA/NICA(0.1) sample after irradiation for 1 h had increased fragility and could not be manipulated with tweezers as a single piece.

The texture characterization, using the puncture test, was only possible for nonirradiated samples. The firmness (N) of the electrospun materials during the puncture test was as follows: 0.261 ± 0.075 (PLA), 0.371 ± 0.090 (PLA/DIBP(1)), and 0.215 ± 0.029 (PLA/NICA(1)). The data are presented as the mean ± S.D. (n = 6). Unfortunately, the irradiated samples turned out to not be strong enough to be studied using this method.

Interestingly, after 1 h of irradiation, local thinning appears on the fibrils, which is shown by the yellow arrows in Figure 3c. Perhaps, it is a consequence of the annealing effect of UV irradiation, causing the formation of volatile compounds and weight loss. The annealing effect of UV irradiation was also observed when electrospun PLA was irradiated in [7]. Intersections of the fibrils in the electrospun material led to local shading from UV irradiation and the preservation of fibril thickness in other areas, which is also clearly visible in Figure 3c.

**Figure 4:**
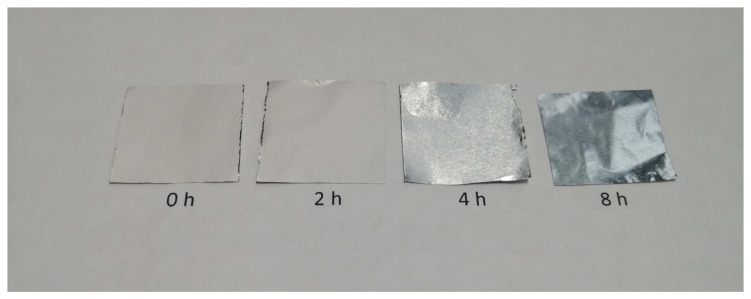
Electrospun PLA samples on aluminum foil irradiated by UV-C for various amounts of time.

Two hours of irradiation resulted in extreme fragility in all the samples of fibrous material; they did not maintain their integrity under any manipulation. After 4 and 8 h of irradiation, the samples lost their fibrous structure and became a sticky transparent layer on the surface of the foil (Figure 4).

### 3.2. Chemical Structure Analysis

**Figure 5:**
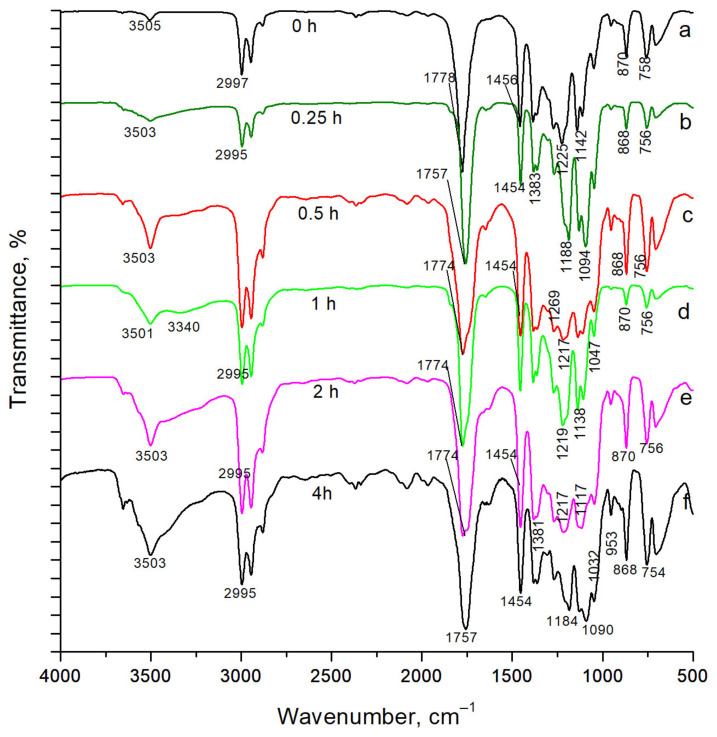
FTIR spectra of PLA after 0–4 h (a–f, respectively) of UV-C irradiation.

The chemical structure of PLA changed upon irradiation in the UV-C range, which was confirmed by comparing the FTIR spectra of the irradiated samples with the spectrum of the unirradiated fibrous PLA material (Figure 5). It is noticeable that irradiation increased the absorption bands of stretching vibrations of hydroxyl groups. The terminal hydroxyl groups in nonirradiated PLA fibers showed a relatively small absorption peak at 3505 cm^−1^. However, with increasing photodestruction time, an increasing number of ester bonds in the polymer chain were broken and, as a result, the number of terminal hydroxyl and carboxyl groups increased, while absorption in the 3600–3100 cm^−1^ region increased significantly.

Table 2 presents the assignment of the main absorption bands in the FTIR spectra of the fibrous PLA samples according to [24]. The FTIR spectrum of the sample irradiated for 0.5 h revealed not only an increase in the absorption intensity of the hydroxyl groups, but also a broadening of the stretching vibration band of the carbonyl groups at 1774 cm^−1^, which is also associated with depolymerization and the appearance of new terminal groups in the polymer [25]. At this irradiation time of 0.5 h, fibers without stabilizer additives began to demonstrate pronounced fragility.

The sample irradiated for 4 h became a sticky film, without a fibrous structure. Its FTIR spectrum (Figure 5f) was characterized by a significant intensity of a group of absorption bands in the range of 3600–3100 cm^−1^ and a band at 1047 cm^−1^, belonging to the stretching and bending vibrations of OH groups, respectively.

**Figure 6:**
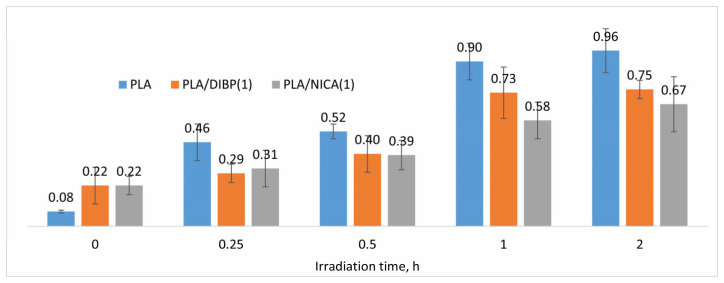
Relative integrated intensities of FTIR absorption at 3600–3100 cm^−1^ for PLA, PLA/DIBP(1), and PLA/NICA(1) at different irradiation times (0–2 h).

The FTIR spectra of the materials with added photostabilizers, PLA/DIBP(1) and PLA/NICA(1), demonstrated the same patterns in the growth of absorption bands upon irradiation. The integration of absorption in the region of 3600–3100 cm^−1^ was carried out and the effect of photodestruction on stabilized and non-stabilized fibers was compared based on the FTIR spectra. In this case, the integrated absorption intensity in the region of 1890–1530 cm^−1^ was chosen as an internal standard. The ratios of the integral intensities of the FTIR spectrum regions 3600–3100 and 1890–1530 cm^−1^ for each irradiation time interval (0–2 h) form the dependences presented in Figure 6, quantitatively reflecting the photodestruction processes taking place in the chemical structure of the polymer. After 0.25 h of irradiation, the photoprotective effect of the studied low-molecular additives became visible, namely the samples with additives showed lower values of relative absorption intensity in the region of 3600–3100 cm^−1^, despite the fact that the nonirradiated fibers with additives had higher absorption values. In Figure 6, a surge in this value for the non-stabilized sample subject to 1 h of irradiation is clearly visible, leading to a sharp increase in the fragility of the fibers. However, the difference in this value for between 1 and 2 h of irradiation was not significant. For example, in [8], a similar sharp increase in the absorption band at 3400 cm^−1^ was observed after 1 day of irradiation of TiO_2_-loaded electrospun fibers of polylactide under UVA light.

It is also noticeable that the additives, DIBP and NICA, slow down the formation of new hydroxyl groups during the destructive processes in the polymer. In the samples irradiated for 1 h, the difference in the content of hydroxyl groups between the stabilized and non-stabilized samples reached a maximum.

**Figure 7:**
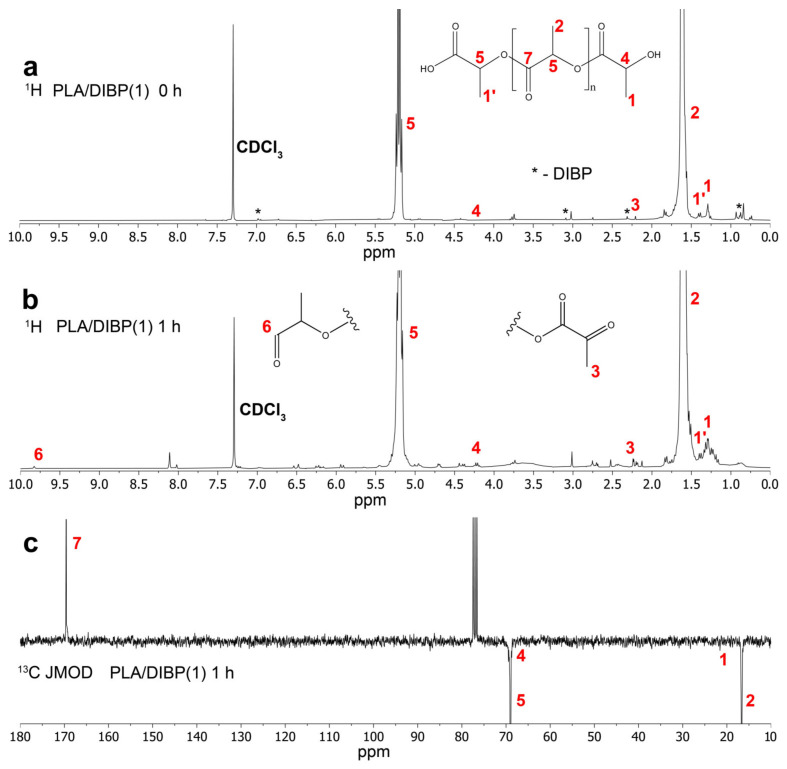
The ^1^H spectra of the original sample (**a**) and 1 h UV irradiated sample (**b**) PLA/DIBP(1); the ^13^C JMOD NMR spectrum of 1 h UV irradiated PLA/DIBP(1) sample (**c**); signals of DIBP are marked with *.

Figure 7 shows the ^1^H spectrum of the non-irradiated PLA/DIBP(1) sample (Figure 7a). The ^1^H and ^13^C JMOD NMR spectra of the samples irradiated for 1 h are presented in Figure 7b,c. The spectrum of the irradiated PLA (Figure 7b) shows increased signals of terminal methyl groups at the chemical shifts of 1.0–1.49 ppm and 2.0–2.5 ppm. The peaks in the region of 4.0–4.5 ppm are presumably associated with the CH of the terminal lactoyl groups. Signals indicating the appearance of new terminal aldehyde groups are found at 9.8 ppm in the ^1^H spectrum.

**Figure 8:**
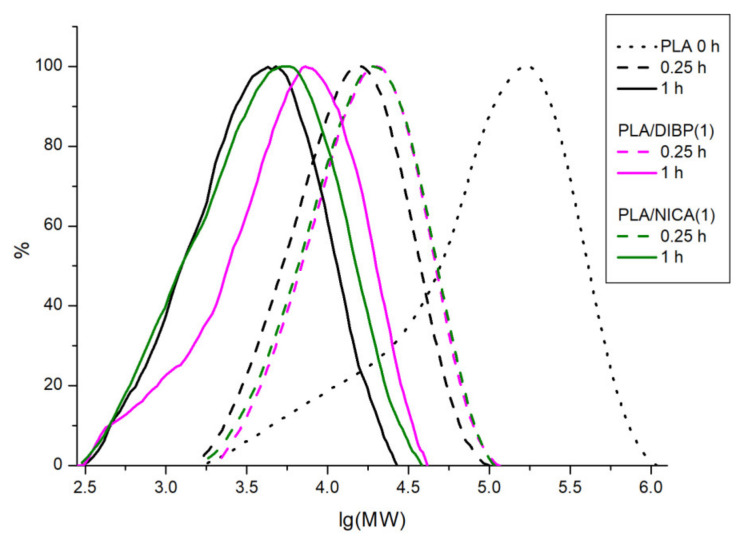
MMD of PLA, PLA/DIBP(1), and PLA/NICA(1) samples.

GPC chromatograms were obtained using a refractometric detector. The resulting molecular mass distribution curves (MMD) are presented in Figure 8. Table 3 shows the results of the number-average (M_n_) and mass-average (M_w_) molecular weight, as well as the degree of polydispersity (D), for the studied samples.

A significant decrease in the molecular weight upon irradiation was noted both for the electrospun material not stabilized by additives and for the samples PLA/DIBP(1) and PLA/NICA(1). This decrease in the molecular weight of the polymer after irradiation with UV-C is associated with a decrease in the number of ester bonds between the PLA monomer units. For comparison, according to data in the literature, an intensity of 2.54 mW/cm^2^ of UV-C at 245 nm led to a decrease in the molecular weight of PLA by more than four times after 24 h of irradiation [13,26].

### 3.3. TG–DSC Analysis

**Figure 9:**
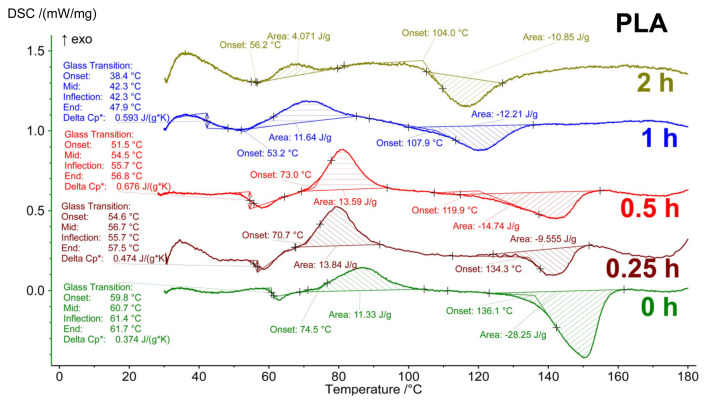
DSC curves of PLA samples without photoprotective additives after different durations of UV-C irradiation.

**Figure 10:**
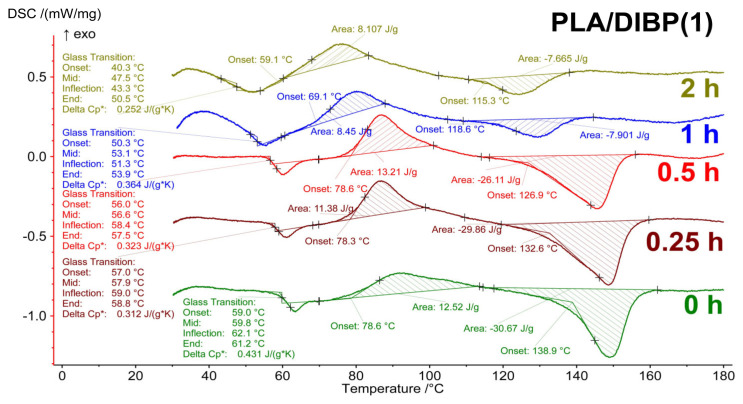
DSC curves of PLA samples with DIBP after different durations of UV-C irradiation.

**Figure 11:**
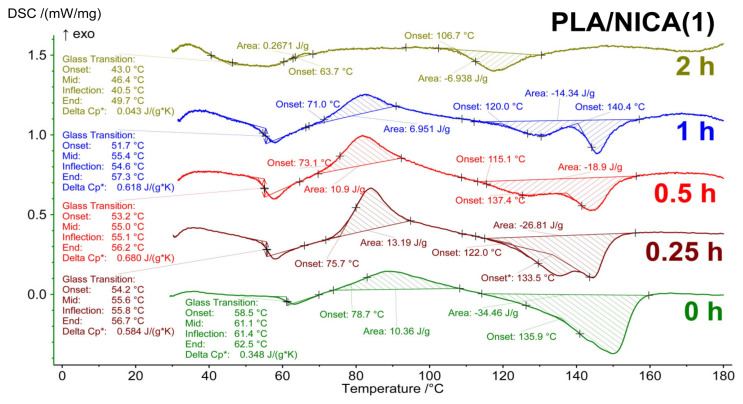
DSC curves of PLA samples with NICA after different durations of UV-C irradiation.

Thermal analysis of the irradiated polylactide samples without additives, and with DIBP and NICA additives, was carried out in an air atmosphere heated to 450 °C. The DSC curves (Figure 9, Figure 10 and Figure 11) for the irradiated and initial samples in the range of 25–170 °C demonstrate a number of phase transitions: a glass transition (T_g_) of about 60 °C, cold crystallization (T_cc_) at about 65–80 °C, and melting (T_melt_) at 110–140 °C. The appearance of a cold crystallization peak means that during the formation of fibers by electrospinning and upon cooling after stabilization at 60 °C, the conditions for crystallization were not achieved, i.e., the polymer structure was in a nonequilibrium state. In [24], the cold crystallization effect was interpreted as the result of incomplete/absent crystallization, with a lack of nucleation during cooling of the PLA.

Table 4 presents the values of the thermal effects on the TG–DSC curves for the material without the addition of stabilizers. The most sensitive indicator to the effect of irradiation was the T_g_ value, which smoothly decreased from 60 °C to 43.3 °C over 1 h of irradiation, along with the fact that the corresponding values of ΔCp* practically did not change until 30 min of irradiation. A sharp drop in ΔCp* was detected after 1 h of irradiation for both the sample without additives (Table 4) and with additives (Figure 9, Figure 10 and Figure 11). In samples irradiated for 2 h or more, signs of the glass transition in the thermograms are no longer present in the sample without additives and with the addition of NICA. However, for the PLA/DIBP(1) sample, it is possible that the glass transition effect remained after two hours of irradiation.

**Figure 12:**
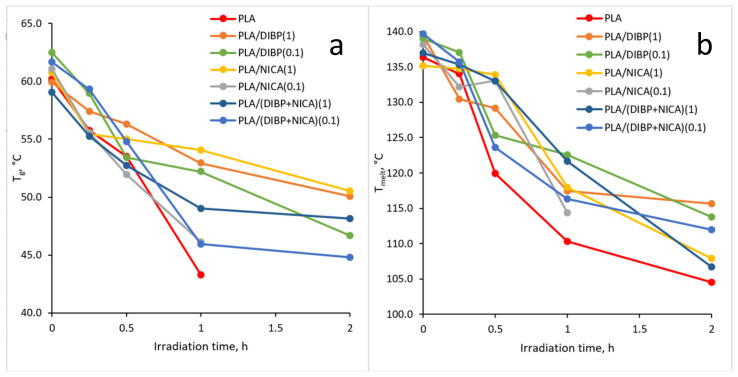
T_g_ (**a**) and T_melt_ (**b**) dependences on the irradiation time of the PLA samples.

In Figure 12, it can be seen that both the T_g_ and other thermal effects in the irradiated samples also change smoothly as a result of irradiation. For example, all values at the beginning of phase transitions shift to the region of low temperatures, and the change in heat capacity, enthalpy of the cold crystallization effect, and melting decrease from less irradiated to more irradiated samples.

The UV protective effect, caused by the introduction of DIBP and NICA additives, is noticeable due to the higher T_g_ temperatures of the samples irradiated for 1 h (Figure 12). Just like with the FTIR analysis, the maximum difference between the T_g_ is observed after 1 h of irradiation.

For example, in [27], when irradiating PLA films that were 10 µm thick, which is 20 times thicker than the fibers studied here, a significant difference in the T_g_ of nano-silver stabilized polylactide began to be observed after 20 h of UV-C irradiation.

When the stabilized fibers were irradiated, signs of the glass transition phase transition were reliably identified even after 2 h of irradiation, except in the case of the sample with the lowest concentration of NICA, namely 0.06%, when no T_g_ effect was detected in a sample irradiated for 2 h.

**Figure 13:**
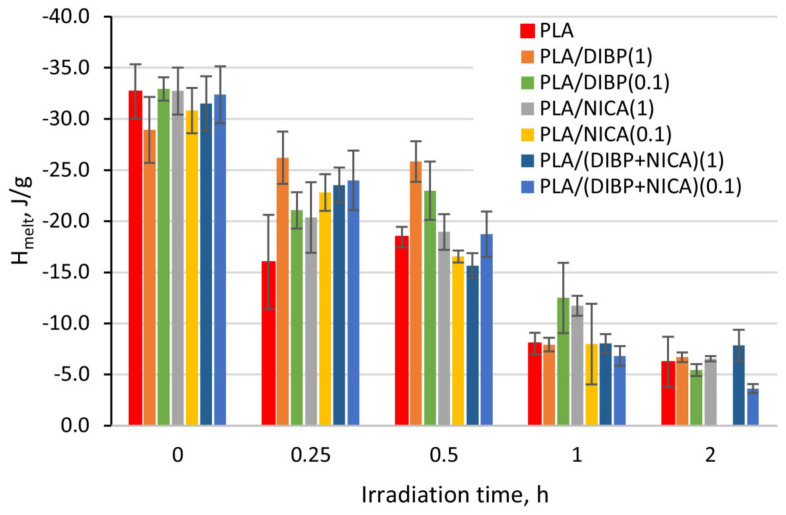
Values of melting enthalpy of PLA samples, depending on irradiation time.

The decrease in the T_g_ during irradiation indicates that the polymer structure becomes less integrated, and the internal volume of the polymer increases, which is facilitated by an increase in the proportion of low molecular weight compounds formed. In this regard, it should be noted that the introduction of DIBP or NICA in various quantities has virtually no effect on the T_g_, T_cc_, and T_melt_ of the nonirradiated samples. It should be noted that the melting peak of 136.3 °C was observed not only for the initial PLA, but also at up to 2 h of irradiation, although gradually decreasing in regard to the enthalpy value. A sharp decrease in the enthalpy of melting and the disappearance of the melting peak was observed after 2 h of irradiation (Figure 13), which means greater changes in the PLA structure than that revealed in [18,28] with the same UV-C wavelength, but where the PLA was tested in the form of films. In those works, this peak disappeared after more than 4 h of UV-C exposure. In [29], there was a sharp decline in the value of the energy of melting after only 15 min of irradiation. Using ZnO as a photoprotector, the greatest difference compared to unprotected PLA fibers was detected after 4 h of irradiation. During photodecomposition of PLA with polybutylene adipate terephthalate as a photoprotector at 254 nm in [30], the T_g_ shifted to higher values, while the melting temperature decreased by several degrees.

The PLA/DIBP(1) and PLA/NICA(1) samples showed the best results in regard to protecting the PLA from UV-C after 1 and 2 h of irradiation, based on the T_g_ values. For example, in [28], a noticeable photoprotective effect after 2 h of irradiation appeared when 3% or 5% of modified montmorillonite are introduced into polylactide.

In [31], synergistic effects of photostabilization between UV absorbance and the antioxidant properties of polyphenols of plant extracts were noticed. However, DIBP and NICA were not very effective when used together, despite the shielding function of NICA and the antioxidant properties of DIBP. The analysis of the irradiated PLA/(DIBP + NICA)(0.1) sample revealed a significant decrease in the T_g_ at 1 and 2 h of irradiation (Figure 12a). The PLA/(DIBP + NICA)(1) sample showed a T_g_ at 5 °C less at 1 h of irradiation, which is noticeably worse in comparison with the PLA/DIBP(1) and PLA/NICA(1) samples; although, the total molar concentration of additives was equal in these samples.

**Figure 14:**
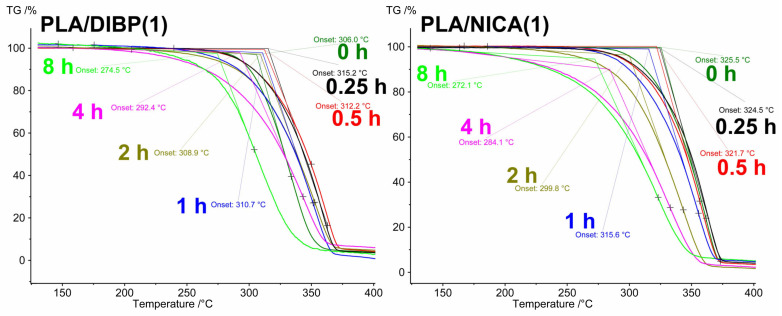
Thermal destruction of PLA samples with DIBP and NICA additives after 0–8 h of irradiation.

The polymer structure of nonirradiated electrospun PLA material undergoes thermal–oxidative destruction at 319.5 ± 0.6 °C, as determined by the beginning of a decrease in the mass on the TG curve. The duration of irradiation led to a decrease in the temperature at the onset of PLA decomposition, from 319.5 °C to 260.3 °C after 8 h of irradiation (Figure 14). The addition of 1 and 0.1 wt.% of DIBP decreased the onset temperature of PLA decomposition by 15.8 °C and 10.4 °C, respectively. The addition of NICA, on the contrary, promoted thermal stability of the polymer and increased the onset temperature of PLA decomposition by 6.3 °C in the PLA/NICA(1) sample and by 2.5 °C in the PLA/NICA(0.1) sample (Figure 14). However, both NICA and DIBP showed a more or less pronounced thermoprotective effect on the irradiated samples, which can be explained by the better preservation of the polymer structure of the fibers modified with additives after irradiation compared to fibers without additives.

In [32], 0.8% of UV absorption groups of benzophenones in the PLA structure had a similar effect on the temperature of thermal decomposition in the samples irradiated for 2 h, namely a decrease in temperature by 7.2 °C, which is comparable to our results.

### 3.4. Py–GC/MS Analysis

**Figure 15:**
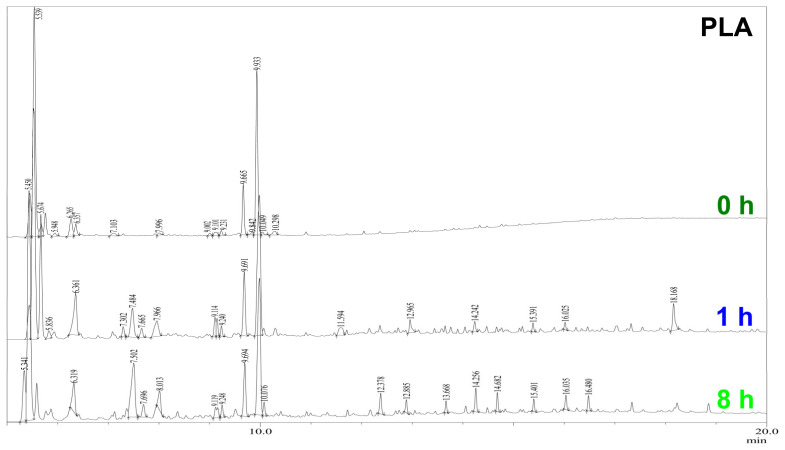
Pyrolysis chromatograms of PLA samples without photoprotective additives after different durations of UV-C irradiation.

The Py–GC/MS chromatograms (Figure 15) of the nonirradiated samples have a set of peaks in regard to substances typical of the pyrolysis decomposition of PLA. The most intense peaks in the chromatograms are the peaks of carbon dioxide, acetaldehyde, and lactides (see Table 5).

When comparing the chromatograms of pyrolysis products of nonirradiated and irradiated PLA materials, it is noticeable that the most obvious changes appear in the RT range from 7.4 min to 8 min with increasing irradiation time, namely the intensity and number of peaks in this range increases. Analysis of the chromatograms showed that the relative total content of methyltartronic and 2-methyl-2-butenoic acids in pyrolysis products increased approximately 20 times after 1 h of UV-C irradiation (Figure 15, 1 h) and another 1.5 times over the next 7 h of irradiation (Figure 15, 8 h).

**Figure 16:**
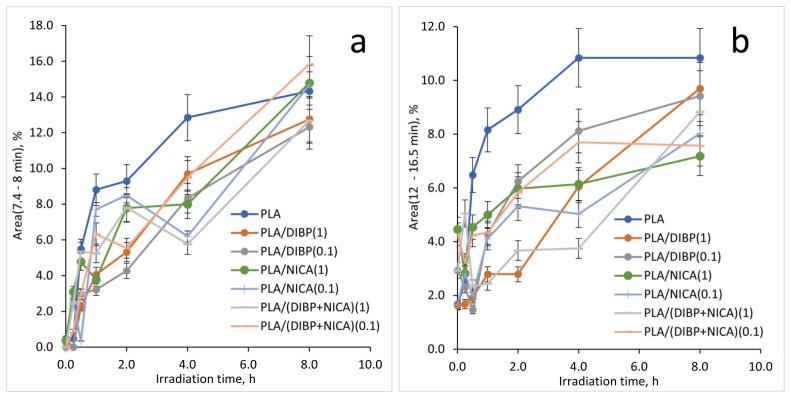
Yield of pyrolysis products with RT from 7.4 min to 8 min (**a**), and RT from 12 min to 16.5 min (**b**) for PLA samples depending on the irradiation time.

In the RT region, from 12 min to 16.5 min, in the chromatograms (Figure 15) of irradiated PLA materials, there is an increase in the number and intensity of the peaks corresponding to two and three basic acids and their esters (C5–C8), with molecular weights from 146 to 246. Therefore, these groups of peaks were used to control the effectiveness of the photoprotective effect of NICA and DIBP. More detailed changes in the amounts of these pyrolysis products during irradiation, as well as the effect of the addition of photostabilizers, NICA and DIBP, were analyzed by plotting the dependence of the yield of pyrolysis products with the RT from 7.4 min to 8 min, and with the RT from 12 min to 16.5 min (Figure 16) on the irradiation time.

The amount of acidic pyrolysis products with an RT from 7.4 min to 8 min (Figure 16) increases very quickly up to 1 h of irradiation; after 4 h of irradiation, the curve reaches a plateau with a slight increase. Acidic products may indicate autohydrolysis, explaining the abrupt transformation from a brittle to a sticky material. The polymer materials with NICA and DIBP additives showed a decrease in the yield of methyltartronic and 2-methyl-2-butenoic acids in pyrolysis products in the irradiation time range from 1 to 4 h, which indicates the preservation of the protective effect of these stabilizers at 4 h of irradiation. With 1 h irradiation, the greatest difference in the yield of these acids relative to pure PLA was shown by: PLA/DIBP(1), PLA/NICA(1), and PLA/DIBP(0.1). By 8 h, no significant difference was observed in the stabilized samples and the comparison sample.

The appearance of products in the RT region from 12 min to 16.5 min (Figure 16) also depended on the photoprotector introduced into the polymer. Among the samples irradiated for 1 and 2 h, the samples PLA/DIBP(1) and PLA/(DIBP + NICA)(1) were different for the better: the yield of these thermal destruction products was the lowest.

### 3.5. Cell Morphology

The application experiments on the biocompatibility of the electrospun PLA sample were carried out using the cell adhesion, morphology, proliferation, and viability of human fibroblast to demonstrate the practical utility of the material. The viability of the cells was studied after cultivation on the surface of the electrospun PLA (s-P), under the electrospun PLA on the surface of culture plastic (u-P), on the surface of electrospun PLA applied to foil (s-P-F), and under electrospun PLA applied to foil on the surface of culture plastic (u-P-F).

**Figure 17:**
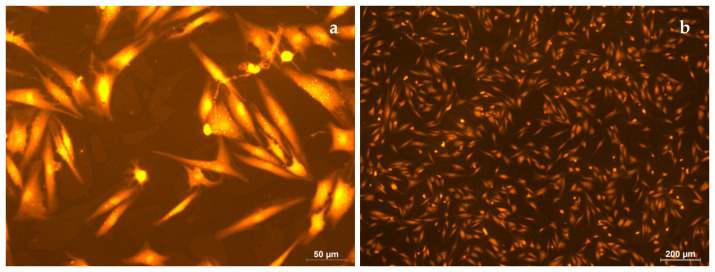
Human fibroblasts after 5 days of cultivation under standard conditions. Magnification 50× (**a**) and 200× (**b**), staining with rhodamine.

In the control experiment, after 5 days of incubation, all the cells had an elongated shape (Figure 17a). The cell length was 109 ± 18 µm (Figure 17b).

**Figure 18:**
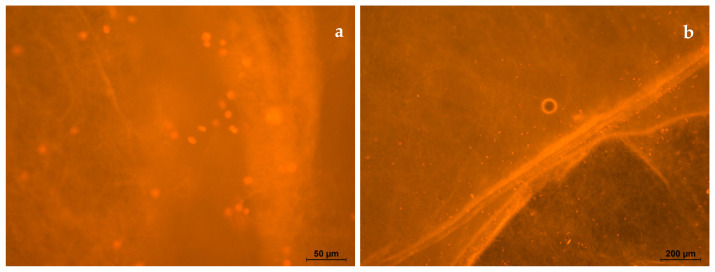
Human fibroblasts after 5 days of cultivation on s-P. Magnification 50× (**a**) and 200× (**b**), staining with rhodamine.

**Figure 19:**
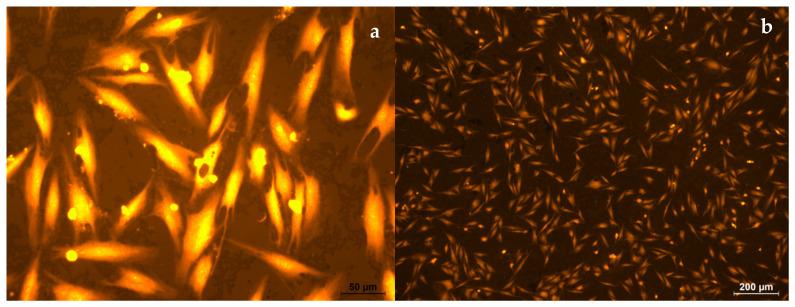
Human fibroblasts after 5 days of cultivation on u-P. Magnification 50× (**a**) and 200× (**b**), staining with rhodamine.

On the surface of the electrospun PLA (s-P), all the cells are spherical in shape, and the cell diameter is 11 ± 2 µm (Figure 18a,b). The cells located under the electrospun PLA, on the surface of culture plastic (u-P), had normal morphology, with developed filamentous and lamellar pseudopodia (Figure 19a,b). The cell length was 91 ± 17 µm, which was comparable to the control.

**Figure 20:**
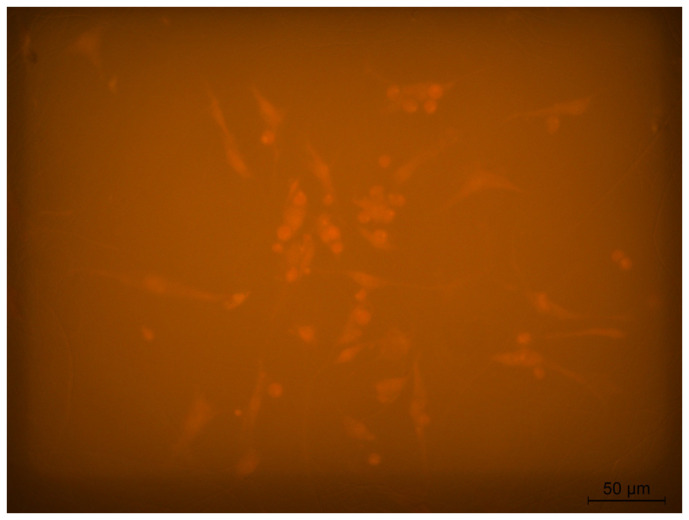
Human fibroblasts after 5 days of cultivation on s-P-F. Magnification 50×, staining with rhodamine.

The cultivation of fibroblasts on the surface of the electrospun PLA applied to foil (s-P-F) showed that cells adhered to the surface of the biomaterial. However, only some of the cells were in the growth stage; the length of these cells was 60 ± 18 µm. Compared to the control, on s-P-F, the cell growth rate slowed down by 40%. The other part of the cells (approximately 50%) had a spherical shape, with a diameter of 11 ± 3 μm (Figure 20); apparently, these cells were not viable. Thus, s-P-F has greater biocompatibility compared to s-P, however, it is insufficient for the normal functioning of cells.

**Figure 21:**
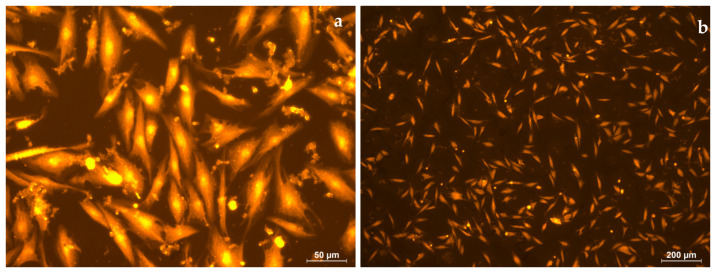
Human fibroblasts after 5 days of cultivation on u-P-F. Magnification 50× (**a**) and 200× (**b**), staining with rhodamine.

The morphometric assessment of the fibroblasts cultured on culture plastic, in the presence electrospun PLA on foil (u-P-F), showed that u-P-F does not have an inhibitory effect on cell viability (Figure 21a,b). The cell length was 83 ± 9 µm, which was comparable to the control values.

These results indicate that electrospun PLA does not have a toxic effect on human fibroblasts, but the surface properties (physicochemical, structural, and mechanical) can prevent cell growth and proliferation. The u-P and u-P-F samples showed the absence of an inhibitory effect on cell viability and had a morphology closer to the control. The identified patterns in the biocompatibility of the electrospun PLA can be used in the development of wound dressing systems.

## 4. Conclusions

In this work, we have quantified the effectiveness of DIBP and NICA, the new photostabilizers for PLA materials against UV-C (254 nm) radiation. The performance of the tested stabilizers was close to the literature data on natural and biocompatible substances, which are currently most relevant for creating PLA composites with a variety of functions. The addition of 26.3 × 10^−3^ mmol/g of DIBP or NICA, as well as combinations of DIBP and NICA, to the polymer preserved the elasticity and strength of the electrospun PLA samples irradiated for 30 min. FTIR spectroscopy revealed an increase in absorption in the region of 3600–3100 cm^−1^ of the spectra of stabilized and non-stabilized PLA fibers after irradiation. In the samples irradiated for 1 h, the difference in the content of hydroxyl groups between the stabilized and non-stabilized samples reached a maximum. The glass transition (T_g_) remained after 2 h of irradiation when using the photostabilizers DIBP and NICA and their combinations. The PLA/DIBP(1) and PLA/NICA(1) compositions showed the best results in protecting PLA from UV-C radiation based on the T_g_ values; although, the combinations of DIBP and NICA were not as effective.

The Py–GC/MS analysis demonstrated an increase in the content of methyltartronic and 2-methyl-2-butenoic acids in the pyrolysis products after 1 h of UV-C irradiation, as well as an increase in the content of heavy fragments, with molecular weights from 146 to 246 and with large retention times. Polymer materials with the addition of DIBP and NICA showed a decrease in the yield of methyltartronic and 2-methyl-2-butenoic acids in the pyrolysis products in the irradiation time range from 1 to 4 h, which indicates that the protective effect of these stabilizers was retained after 4 h of irradiation. Considering the higher photoprotective activity of DIBP, it can be assumed that terpenophenols synthesized from natural precursors can have an application as a bio-friendly alternative to synthetic photostabilizers.

## Figures and Tables

**Table 1:** Composition of PLA/DIBP, PLA/NICA, and PLA/DIBP/NICA samples.

Sample	Additive Concentration, mmol/g	Mass Fraction of Additive, % *w*/*w*
PLA/DIBP(1)	26.3 × 10^−3^	1%
PLA/DIBP(0.1)	2.6 × 10^−3^	0.1%
PLA/NICA(1)	26.3 × 10^−3^	0.6%
PLA/NICA(0.1)	2.6 × 10^−3^	0.06%
PLA/(DIBP + NICA)(1)	13.15 × 10^−3^ (DIBP) + 13.15 × 10^−3^ (NICA)	0.5 (DIBP); 0.3 (NICA)
PLA/(DIBP + NICA)(0.1)	1.31 × 10^−3^ (DIBP) + 1.31 × 10^−3^ (NICA)	0.05 (DIBP); 0.03 (NICA)

**Table 2:** Assignment of absorption bands in FTIR spectra of PLA.

Wave Number, cm^−1^	Band Assignment
3600–3100	stretch vibrations –OH
2943; 2995	stretch vibrations –CH–
1774	stretch vibrations –C=O
1454	deformation –CH_3_
1381; 1361	deformation –CH–
1219	deformation –C=O
1200–1100	stretch vibrations –C–O–
1047	deformation –OH
953; 868	stretch vibrations –C–C–

**Table 3:** Results of determination of molecular weights.

Sample	Time of UV-C Irradiation, min	M_n_, Da	M_w_, Da	D
PLA	0	51,000	168,000	3.3
PLA	15	11,000	20,000	1.7
PLA/DIBP(1)	15	14,000	24,000	1.7
PLA/NICA(1)	15	14,000	24,000	1.7
PLA	60	2700	5500	2.0
PLA/DIBP(1)	60	3900	9200	2.3
PLA/NICA(1)	60	3000	6900	2.3

**Table 4:** Thermal effects of the samples without additives.

Irradiation Time	T_g_, °C	ΔCp*, J/(g·K)	T_cc_, °C	H_cc_, J/g	T_melt_, °C	H_melt_, J/g	T_degr_, °C
0 h	60.2 ± 0.5	0.50 ± 0.06	73.7 ± 1.1	12.5 ± 2.2	136.3 ± 0.5	−32.7 ± 2.7	319.5 ± 0.6
0.25 h	55.7 ± 1.6	0.52 ± 0.10	69.1 ± 1.4	11.0 ± 2.4	134.0 ± 1.3	−16.0 ± 4.6	316.0 ± 0.9
0.5 h	53.5 ± 1.3	0.60 ± 0.04	73.3 ± 0.3	11.2 ± 3.4	119.9 ± 1.1	−18.4 ± 1.0	310.5 ± 0.8
1 h	43.3 ± 1.0	0.12 ± 0.05	53.2 ± 1.6	8.8 ± 2.9	110.3 ± 0.6	−8.0 ± 1.1	304.2 ± 1.1
2 h	-	-	57.3 ± 0.6	5.6 ± 2.1	104.5 ± 1.8	−6.2 ± 2.5	286.9 ± 7.6
4 h	-	-	-	-	-	-	266.9 ± 2.4
8 h	-	-	-	-	-	-	260.3 ± 1.7

**Table 5:** Assignment of PLA pyrolytic degradation products in chromatograms.

№	Substance	Retention Time (RT), min	0 h, %	1 h, %	8 h, %
1	Carbon dioxide	5.45	8.95	4.35	6.9
2	Acetaldehyde	5.539	44.31	35.71	35.39
3	Propanoic acid, 2-oxo-, ethyl ester	5.674	2.45		
4	Acetic acid	5.836		1.03	
5	2-Butanone	5.948	0.7		
6	Propanoic acid	6.265	3.81		
7	Butanedioic acid	6.319			3.82
8	2,3-Pentanedione	6.357	2.11	6.99	
9	3-Hexanone	7.103	0.88		
10	1-Butene-3-ethoxy	7.302		0.92	
11	Methyltartronic acid	7.484		4.48	10.59
12	2-methyl-2-butenoic acid	7.665		1.08	1.56
13	2-methyl-2-butenoic acid	7.996	0.46	3.25	2.18
14	2,5-Furandione, 3,4-dimethyl-	9.002	0.33		
15	Ethene, ethoxy-	9.101	0.85	1.7	1.74
16	Ethene, ethoxy-	9.114		1.39	
17	Propiolactone	9.231	0.74	0.87	1.06
18	1,4-Dioxane-2,5-dione, 3,6-dimethyl-, (3S-cis)-(Meso-lactide)	9.665	7.14	6.66	5.51
19	1,4-Dioxane-2,5-dione, 3,6-dimethyl-, (3S-cis)-	9.842	0.58		
20	1,4-Dioxane-2,5-dione, 3,6-dimethyl-, (3S-cis)-(L-lactide)	9.933	25.08	23.41	20.43
21	Propanoic acid, 2-(methoxymethoxy)-	10.049	0.57		1.13
22	Succinic anhydride	10.298	1.04		
23	Propanoic acid, 2-hydroxy-, butyl ester	12.378			1.82
24	2-Oxopentanedioic acid	12.885			0.95
25	Succinic acid, 2-butoxyethyl ethyl ester	13.668			0.8
26	Propanoic acid, 2-methyl-, 2-ethyl-3-hydroxyhexyl ester	14.256		0.9	1.81
27	Succinic acid, 2-butoxyethyl ethyl ester	14.682			1.29
28	Succinic acid, 2-butoxyethyl ethyl ester	15.401		0.57	0.84
29	Propanoic acid, 2-methyl-, 2-ethyl-3-hydroxyhexyl ester	16.035		0.49	1.09
30	1,1,2-Ethanetricarboxylic acid, triethyl ester	16.48			1.11

## Data Availability

The raw data required to reproduce these findings can be shared. Readers are encouraged to communicate with the corresponding author for more information.
